# A geometrical model of cell fate specification in the mouse blastocyst

**DOI:** 10.1242/dev.202467

**Published:** 2024-04-22

**Authors:** Archishman Raju, Eric D. Siggia

**Affiliations:** ^1^ Simons Centre for the Study of Living Machines, National Centre for Biological Sciences, Tata Institute of Fundamental Research, Bangalore 560065, India; ^2^ Center for Studies in Physics and Biology, Rockefeller University, New York, NY 10065, USA

**Keywords:** Waddington landscape, Dynamical systems, Mouse blastocyst

## Abstract

The lineage decision that generates the epiblast and primitive endoderm from the inner cell mass (ICM) is a paradigm for cell fate specification. Recent mathematics has formalized Waddington's landscape metaphor and proven that lineage decisions in detailed gene network models must conform to a small list of low-dimensional stereotypic changes called bifurcations. The most plausible bifurcation for the ICM is the so-called heteroclinic flip that we define and elaborate here. Our re-analysis of recent data suggests that there is sufficient cell movement in the ICM so the FGF signal, which drives the lineage decision, can be treated as spatially uniform. We thus extend the bifurcation model for a single cell to the entire ICM by means of a self-consistently defined time-dependent FGF signal. This model is consistent with available data and we propose additional dynamic experiments to test it further. This demonstrates that simplified, quantitative and intuitively transparent descriptions are possible when attention is shifted from specific genes to lineages. The flip bifurcation is a very plausible model for any situation where the embryo needs control over the relative proportions of two fates by a morphogen feedback.

## Teaser

**Highlighted Article:** Analysis of recent live-imaging data of ERK signaling in the mouse blastocyst suggests a new model for cell fate specification with a spatially uniform FGF signal that allows extending the Waddington landscape of a single cell to the entire inner cell mass.

## INTRODUCTION

One of the hallmarks of development is the robust allocation of fates, which is tolerant both to noise and external insults. This is often represented as Waddington's metaphor of flow down a landscape, where lineage decisions correspond to valleys splitting (Waddington, 1957). This metaphor has recently been formalized into a mathematical tool for constructing landscapes and fitting cell fate data (Rand et al., 2021).

The pre-implantation mouse blastocyst provides an ideal example of robust allocation of fates because it is experimentally amenable to *ex vivo* manipulation, and is highly reproducible and extensively studied (Simon et al., 2018). The early development of the mouse embryo is relatively well understood and takes place through two binary decisions (Schrode et al., 2013). First, the blastomeres differentiate into the inner cell mass (ICM) and trophectoderm (TE). This is followed by the differentiation of ICM into epiblast (Epi) or primitive endoderm (PrE), which happens between E3.25 and E4. The epiblast becomes the embryo proper with the PrE making supporting tissue. The FGF signaling pathway is known to regulate the second of these two transitions, although the exact mechanism through which it acts is still under debate (Kang et al., 2013; Chowdhary and Hadjantonakis, 2022). Two receptors respond to the FGF ligand, FGFR1 is present in all ICM cells and FGFR2 is present in PrE only, but both contribute to the lineage decision and partially compensate one another (Molotkov et al., 2017; Kang et al., 2017). The receptors activate a downstream MAP kinase pathway and ERK signaling. Cell fates are usually tracked through the activity of two transcription factors: NANOG, which is required for specification of Epi; and GATA6, which is required for specification of PrE (Chazaud et al., 2006; Plusa et al., 2008).

Our present study is motivated by a set of recent experiments that measured the dynamics of ERK levels through a kinase translocation reporter (KTR). The reporter acts as a substrate for the ERK and localizes from the nucleus to the cytoplasm when ERK is activated. Thus, the cytoplasm to nuclear (C:N) ratio of the reporter acts as a proxy for the ERK activity. This in turn, is a proxy for the level of the FGF signal. The experiments by Simon et al. (2020) took two sets of measurements. First, they measured the ERK activity for a 2 h period at intervals of 5 min during the critical period for fate specification between E3.25 and E3.75. They observed a considerable amount of heterogeneity in the ERK levels but found mean ERK levels over the 2 h period to be substantially higher in the prospective PrE cells than the Epi cells by staining fixed cells for NANOG and GATA6 at the end of the live reporter measurement. Second, to check the consistency of their short term results, they took some long-term measurements of ERK activity in the early blastocyst (∼E3.25) for a 12 h period with a 15 min interval for measurement. [A contemporaneous study (Pokrass et al., 2020), with no direct relevance for us, found a correlation between the ERK activity in the hour following mitotic exit and lineage selection].

Previous theoretical studies have mathematically modeled the blastocyst using a spatially localized range of FGF signaling and a model of the gene regulatory networks. The most extensive of these models is by Tosenberger et al. (2017), which proposes a gene regulatory circuit with tristability and is consistent with available experimental data perturbing FGF levels by adding it to the media or drug inhibition (Yamanaka et al., 2010). Another study modeled the ICM as a bistable system, and compared this mathematical model to experiments showing that the embryo could maintain a robust composition of cells even when lineage-restricted cells were added or existing specified cells were ablated *in vivo* (Saiz et al., 2020). Another recent model proposed a collective transition where bistability in the system is the result of a pitchfork bifurcation governed by the number of cells (Stanoev et al., 2021).

We propose a compact ‘geometric’ model for this transition that follows Rand et al. (2021). We reduce the number of variables to the mathematical minimum, show that some earlier models relied on parameter tuning, and expose the common features of the ICM transition to other examples of the flip bifurcation. We rigorously extend the landscape metaphor, generally construed as a single cell, to the entire ICM. The key is a re-analysis of the data from Simon et al. (2020), which argue that cells in the ICM are sufficiently mobile so they see a spatially averaged, but time dependent, level of FGF.

Geometric models exploit the parallel phenomenology between experimental embryology and dynamical systems theory. Cell fate specification relies on a gene regulatory network that can be modeled as a set of differential equations. Motion in that space can be thought of as a ‘flow’. Stable fixed points (henceforth known as ‘fixed points’) attract all near by-points. The fixed points are where the flow stops (or is committed to a valley whose subsequent development is not followed). Fixed points correspond to cell types at the relevant timescales. Saddle points, which have both stable and unstable directions, are where the cell makes a decision. Geometrical models focus on the topology of the flows between the saddles and fixed points (Corson and Siggia, 2017; Rand et al., 2021; Sáez et al., 2022a,b; Raju and Siggia, 2023). They model the dynamics in a low-dimensional abstract space, which is to be interpreted as capturing the important part of the full gene expression dynamics. Crucially, cell fates are modeled but not the genes themselves.

We consider two geometrical models for how the transition from ICM to Epi or PrE could take place. We compare the two models to elucidate the difference in their predictions for this fate decision. We show how our preferred geometrical model is consistent with existing experimental data of FGF perturbations and the robust allocation of cell fates in the face of insults. Finally, we discuss how more controlled dynamic experiments can decisively distinguish between alternate geometries.

## RESULTS

### Analysis of spatial correlations in ERK activity

We first analyzed the live imaging data of ERK activity from Simon et al. (2020) to measure spatial correlations in the ERK activity, as a proxy for the FGF. If there are very local cell-cell interactions mediated by the FGF, then we expect the presence of strong spatial correlations in the ERK signal. Alternatively, if cell movement and diffusion of FGF homogenizes the FGF concentrations, then we expect very little spatial correlation in ERK activity.

The first fate decision is between ICM and TE cells. These two sets of cells are spatially separated and manually labeled in the data. We remove the data corresponding to cells that have already specified to TE and focus only on the ERK activity in the ICM cells.

**Fig. 1.**
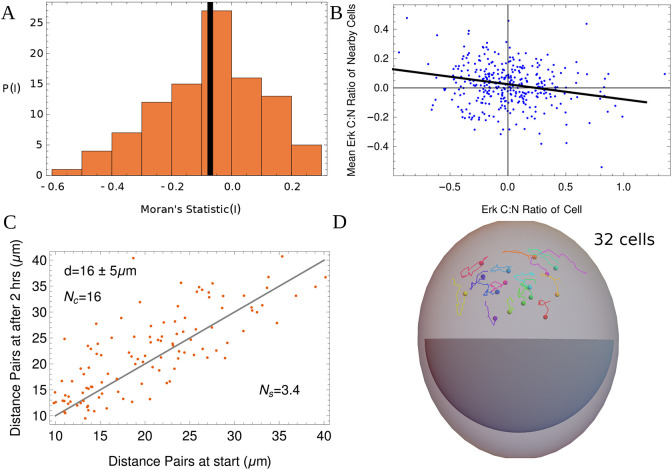
**Analysis of correlations of live-imaging data of ERK activity.** This figure examines the data in Simon et al. (2020). (A) A histogram of the Moran's I index, which is a measure of spatial correlations, with −1 for fully anti-correlated and 1 for fully correlated data. The index is calculated by looking at snapshots separated by 30 min. The ERK activity (C:N ratio) of every cell in 25 embryos is correlated with that of its nearby cells (within a distance of 20 μm). A small negative mean correlation (solid black line) is obtained, equal to −0.07. This is significantly different from 0 (*P*=0.0001) but small compared with −1. (B) A plot of the ERK activity alongside the average value for its neighbors by taking one random snapshot from all 25 embryos. Each embryo has an average of about 14 cells [excluding trophectoderm (TE) cells] for a total of 346 cells used in the plot. The best fit line is has a slope of −0.1, reflecting small but non-zero anti-correlation. The points shown are centered around the mean. (C) A plot of the change in distance between pairs of cells (excluding TE cells) for one embryo over a period of 2 h. The gray line is drawn as a guide and has slope of 1. The number of cells *N_c_*=16. The mean distance between neighboring cells (in the Voronoi sense) d=16±5 μm. As the cells move around, the Voronoi pair contacts change. The number of unique pair contact changes divided by *N*_***c***_ gives the average number of unique neighbor changes per cell: *N*_***s***_=3.4. (D) A representative set of trajectories of 16 ICM-derived cells from one embryo at the 32-cell stage is shown for the duration of 2 h. The outer dome is the minimum bounding ellipsoid of all trajectories, including the trophectoderm (TE) cells that have already segregated. The center of all the cell positions is kept fixed in time. The region marked in dark gray roughly shows the position of the cavity. Different colors represent different cells; spheres show the starting point of the trajectory.

To estimate the effect of the cell movement, we define neighbors based on contacts in a Voronoi tessellation of the cells derived from a nuclear marker. We then track the number of neighbors lost and gained during the 2 h interval of the measurement Figs S1, S2. We estimate that a neighbor is lost or gained on average every 30 min (see Materials and Methods). We show a sample embryo with pair separations and cell movements over the 2 h interval in Fig. 1C,D.

We measure the spatial correlations in the data of Simon et al. (2020) by looking at snapshots of the embryos and then seeing how correlated the ERK activity is. To obtain independent samples, we only take snapshots that are separated by 30 min in the same embryo, to account for the timescale at which cells lose or gain a neighbor.

We use the Moran's I statistic that tests the significance of correlation between a cell and its nearest neighbors (see Materials and Methods). A value of − 1 for the statistic indicates perfect anti-correlation, a value of 1 indicates perfect correlation and 0 implies no correlation.

Fig. 1A is a histogram of the I values across the 25 different embryos (with 4 data points per embryo). The mean statistic is − 0.07±0.02. The statistic is significantly different from 0, with a *P*-value of .0001. This implies that there is some statistically significant negative spatial correlation but the mean is small implying the correlation is very weak.

A second measure of correlated ERK activity is to plot the value of normalized ERK activity alongside the average normalized value for the nearby cells Fig. 1B. This plot also indicates the presence of a weak albeit non-zero spatial negative correlation on average. This is in spite of a positive correlation between sisters after division in agreement with Pokrass et al. (2020) (Fig. S4), indicating that cell movement must scramble the positions of daughter cells. There are also no significant correlations between the time series of the ERK activity of a cell and its Voronoi neighbors (Fig. S3).

**Fig. 2.**
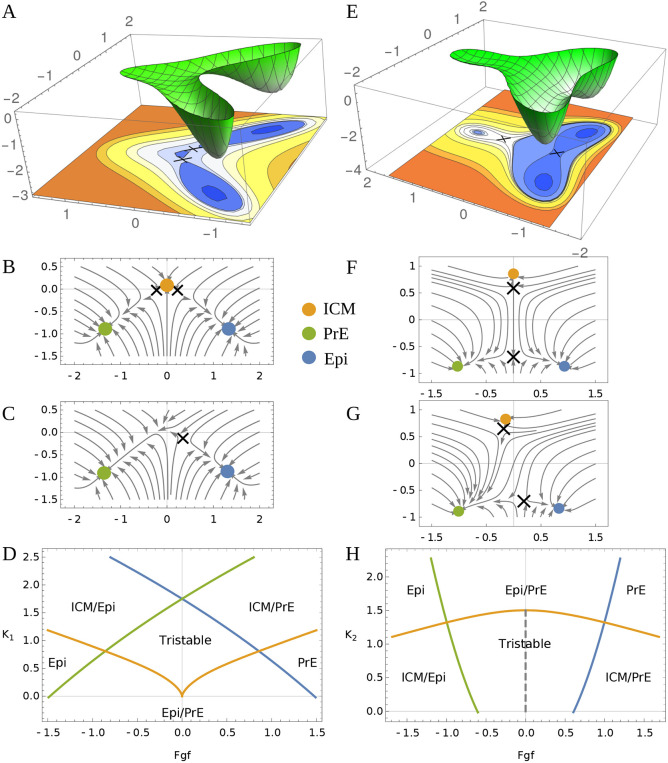
**Two possible geometries for the ICM state near its bifurcation point.** (A) The dual cusp geometry has a potential (schematic) where the central state (ICM) is connected to Epi and PrE but one cannot directly transition from Epi to PrE without passing through the ICM. (B) Flow for the dual cusp (*K*_1_)=0.15. (C) If the FGF concentration is increased, a bifurcation of the ICM state occurs and the flow goes towards PrE (green). (D) The full bifurcation diagram for the dual cusp. Solid lines indicate points of bifurcation where one of the states disappears, with the color indicating which state (*K*_1,2_ controls the tilt of the potential in the vertical direction for each figure). (E) A schematic potential for the heteroclinic flip where Epi and PrE are directly connected. (F) The flow for the heteroclinic flip with *K*_2_=1.4, with the FGF parameter set to the unstable point where the flow from the upper saddle hits the lower one. (G) If the FGF concentration is increased, flows from the saddle near the ICM state go towards PrE but no bifurcation occurs. (H) The full bifurcation diagram for the heteroclinic flip. Solid lines show the point at which bifurcations occur, as in D; the dashed line indicates when the flow out of the upper saddle precisely hits the lower one. The bifurcation of the ICM state takes place at *K*_1_=0 for the cusp and at *K*_2_=1.5 for the flip.

These results all imply that FGF diffusion and cell movement together do not allow the persistence of any strong spatial correlations in ERK activity. Thus, we model FGF as a spatially uniform but time dependent signal that acts equally on all cells. Its value is the sum of contributions from each cell as a function of its internal state, i.e. its position on the landscapes in Fig. 2, the specification of which is an important part of the model.

### Constructing the geometrical model

Our recent work (Rand et al., 2021) strongly suggests two possible geometries for the ICM lineage decision that we model as a tristable dynamic system, with the ICM, Epi and PrE as the three available states. The two geometries are shown in Fig. 2, as landscapes and corresponding flows downhill. The landscapes are described by two parameters: one controls the stability of the ICM state and is primarily a tilt in the vertical direction; the second is the effect of FGF, which tilts horizontally (Fig. 2D,H).

The dual cusp (Fig. 2A,B) is a local bifurcation in that the flow intersecting a small circle around the ICM and two saddle points, denoted as X in Fig. 2B, is identical to that for a circle around the single saddle in Fig. 2C. The dual cusp is a single ‘point’ in the two-dimensional parameter space (the origin [0,0] in Fig. 2D) at which an ICM cell loses stability towards Epi or PrE. The final outcomes may be biased between Epi and PrE, but both are accessible. The dual cusp geometry is effectively one dimensional, all the decisions happen on the flow curve connecting the three states (thus, vertical and horizontal tilts in the landscape may have similar effects). The simple geometric fact that the surrounding flow compresses all points toward a one-dimensional curve with two saddles and three fixed points has profound implications for dynamic experiments, as we elaborate in the following.

In the alternative flip geometry, the ICM loses stability along a ‘line’ in parameter space(Fig. 2H versus D); in this sense does not require parameter tuning, whereas the dual cusp does. (A random line in the parameter space of the flip has a finite probability of destabilizing the ICM, with zero probability of hitting the origin in Fig. 2D.) The flip bifurcation is global and involves two ‘decisions’: the first (local) is whether to destabilize the ICM; the second (global) is whether to flow to PrE or Epi. Both are ‘typical’ in that they occur along a line in parameter space. The dynamics of the global decision can not be reduced to a small region around a saddle point, in contrast to the dual cusp; thus, the cells leaving the ICM sample the topography and integrate the morphogen signals. As we elaborate below, the ICM cells first flow down a confining valley towards the lower saddle point, the valley then opens, becoming a ridge, and the cells flow off the ridge to either PrE or Epi. The lower saddle separating the PrE and Epi is unstable to the slightest amount of noise. The flip geometry is inherently two dimensional and cells tending towards Epi can be diverted to PrE by exogenous FGF without passing through the ICM.

Finally, it's not understood whether the signal that destabilizes the ICM around E3.25 is a reflection of cell number or absolute time, and its molecular identity. However, in a geometric model, all that matters is that the previously stable ICM becomes unstable by a bifurcation with the upper saddle point.

### Quantifying the effect of FGF on a single cell

**Fig. 3.**
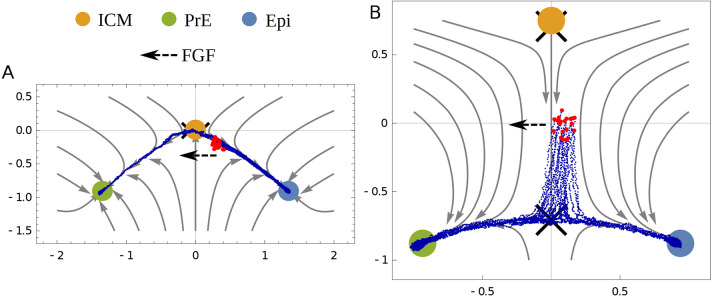
**Transition of fate to PrE with FGF overexpression.** To demonstrate the difference in the two geometries, we show how external FGF changes the fate of cells that are tending towards Epi but not yet committed to that state near the time when the ICM state becomes unstable. Blue lines show trajectories for multiple cells whose initial conditions are shown in red. The dashed arrow shows the direction in which FGF acts. A fraction of the cells commit to PrE. (A) In the dual cusp geometry, going from Epi to PrE requires transiting through the ICM state. (B) In the heteroclinic flip, the cells can directly transition from Epi to PrE.

The two geometries make very different predictions for response to a modest pulse of FGF immediately after the ICM state has bifurcated away (Fig. 3). If the cell is tending towards Epi, then a pulse of FGF under the dual cusp geometry will force the cell to revert to ICM on its way to the PrE state. The meaning of lineage in this context is that no other paths between Epi and PrE exist. For the flip, on the other hand, a direct transition between the two states in possible, more so if the FGF is applied before the wells representing the states are too deep.

**Fig. 4.**
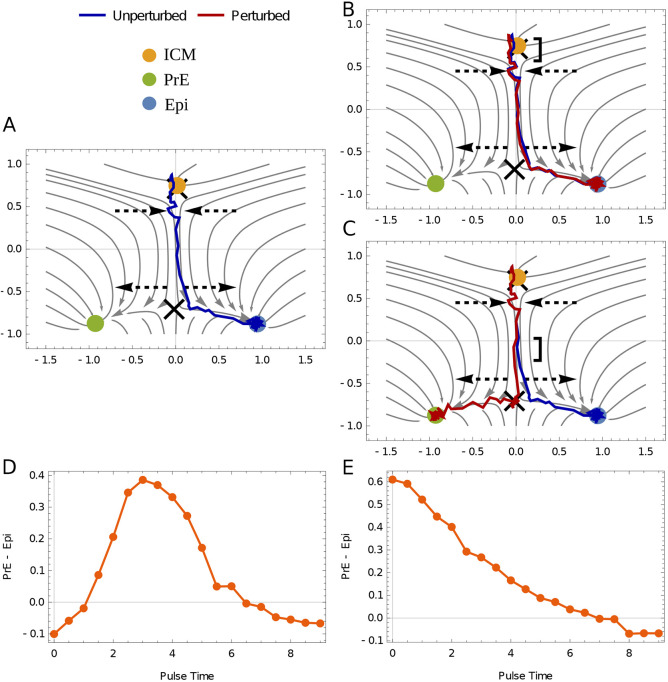
**Time-dependent perturbations differentiate the two geometries.** A standardized pulse of FGF (magnitude 0.2, duration 0.5) is applied at various times to a topography slightly biased to Epi without internal FGF feedback to illustrate the two topologies. (A) A representative trajectory in the absence of a FGF pulse. Dashed arrows indicate regions where the flow converges and diverges, following Fig. 2E. (B) If external FGF is put in early (square bracket), the trajectory (red) deviates slightly but has time to recover and moves back to Epi. (C) If FGF is put in late (square bracket), the diverging flow amplifies the perturbation and the trajectory (red) moves towards PrE. (D) Sensitivity to timed FGF perturbations for the heteroclinic flip calculated as the fraction difference between PrE and Epi. The limits at the beginning and end times show the assumed bias towards Epi (modeled as a constant negative magnitude of 0.004 that is the same for all panels). Initially, a pulse of FGF has little effect but pulses at intermediate times can have large effects. Time is normalized to be between 0 and 10. FGF feedback is not modeled here (see Fig. 5A). (E) Sensitivity to timed FGF perturbations in the dual cusp given a constant bias towards Epi. An initial pulse is strong enough to give a large fraction of PrE but later pulses have no effect, as they are not large enough to remove the potential well around the Epi state.

Timed perturbations would convey the most quantitative information about the structure of the landscape, and we consider again a landscape that is biased towards the Epi state and where the ICM state has just undergone a bifurcation. For the flip, as the cell exits the ICM state (Fig. 4A), the flow is compressed and the state is brought towards the heteroclinic orbit connecting the two saddle points. This is akin to rolling down a valley. Thus, an early pulse of FGF when the cell is in the valley will have little effect (Fig. 4B), whereas a pulse when the cell is just exiting the valley will have maximal effect (Fig. 4C,D). For the dual cusp, the sensitivity decreases monotonically in time (Fig. 4E).

**Fig. 5.**
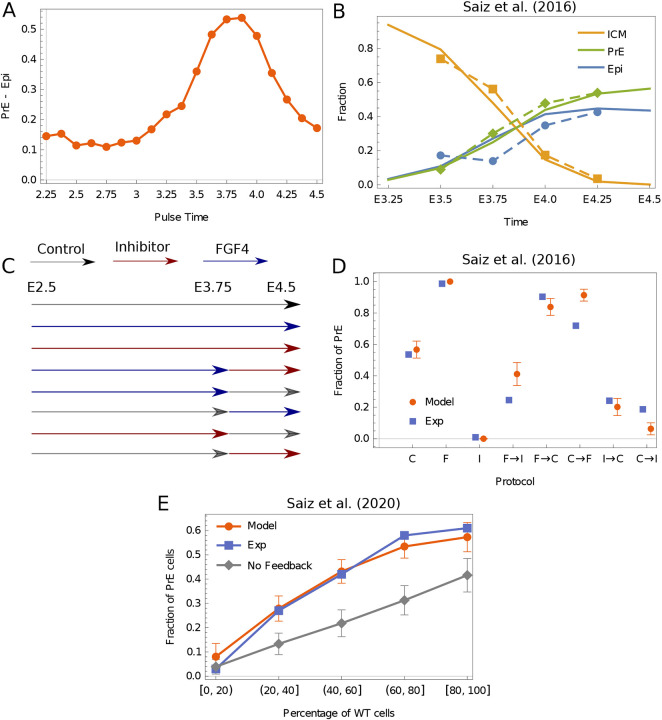
**Theoretical model compared with the results of experiments.** (A) Our model predictions with FGF feedback for the effects of a pulse of FGF (magnitude 0.2, duration 0.125 days) plotted as the fraction difference of PrE and Epi. (B) Experiments from Saiz et al. (2016) estimated the number of ICM/Epi/PrE cells by measuring NANOG and GATA6 expression at different times during the period of specification (dashed lines). Here, we show the analogous results from our model (solid lines). ICM cells were defined as those cells above a certain threshold in the *y* coordinate set to 0.5. Below this, cells were defined either as Epi if *x*>0 or as PrE if *x*<0. We map embryonic time between E2.25 and E4.75 linearly to the time in the model. The bifurcation of the ICM state happens around E3 in the model. (C) Experimental protocols used by Saiz et al. (2016). Embryos were kept in control media, in media with inhibitor and in FGF4-rich media for the length of times indicated. (D) The results of our model for the perturbations in C compared with the experimentally measured results. The horizontal axes are marked by the protocol used: F, FGF4; C, control; I, inhibitor. The model is qualitatively consistent with the data. The media were assumed to be switched exactly at E3.75 and a small uncertainty in the time when media are switched can easily account for discrepancies. The error bars in the theory result from stochastic fluctuations in the model. (E) The results of our model compared with experiments carried out by Saiz et al. (2020). *Gata6^−/−^* cells were combined with wild-type cells in different proportions. The horizontal axis is the percentage of wild-type cells (which are grouped in different brackets). The vertical axis shows the fraction of PrE cells in the terminal state. The gray line shows the results of our model without any FGF feedback, as in Fig. 4. These data therefore set a lower bound on the internal feedback required in the model.

The response of the dual cusp to timed perturbations matches that expected from a naïve model of commitment, i.e. the response decreases as cells commit to a terminal fate. The flip bifurcation has a time at which it is most sensitive because it is global and has to be viewed in two dimensions. Late times are cut off by commitment, as for the dual cusp, whereas early times are restrained by the valley that persists after the ICM destabilizes.

### The geometrical model fits existing experimental data

We fit only the heteroclinic flip geometry to data, as it does not require the parameter tuning of the dual cusp, although we are not aware of any data that explicitly exclude the dual cusp. Under the flip geometry, the dual positive state, NANOG^+^ (Epi) and GATA6^+^ (PrE) signifying ICM, begins as a shallow attractor; however, after it destabilizes, the mutual antagonism between these two states occurs when they fall off the ridge separating the Epi and PrE. (The valley around the unstable ICM persists for some time.) Each cell sees the same potential landscape but evolves with an independent realization of the molecular noise term. There is a common but time-dependent level of FGF, as already illustrated in Fig. 1.

To complete the model, we have to define how the cells produce FGF. Previous models have considered differing molecular mechanisms for how the FGF is controlled by these two transcription factors. Saiz et al. (2020) considered FGF to be activated by NANOG, whereas Tosenberger et al. (2017) considered it to be inhibited by GATA6. By modeling fates only, the geometric model allows us to be agnostic about whether the PrE state represses constitutive FGF expression or whether the Epi state is solely responsible for its production, or both. Similar agnosticism applies to the involvement of other transcription factors that reinforce the lineage decision between Epi and PrE.

In our flow diagrams, the proximity to the two fates is given by the *x* coordinate; +1 is pure Epi and −1 is pure PrE. Experiments indicate that cells that have specified into Epi can trigger the specification of undetermined cells into PrE, but the reverse process does not take place (Saiz et al., 2016). We thus model FGF as proportional to the sum of the *x* coordinates of all cells, ignoring cells for which *x*<0, i.e. only cells near the Epi state produce FGF. A linear dependence on *x* fits existing data, so nothing more elaborate is called for. The effects of this feedback are shown in Fig. 5A for a pulse of FGF at different times. It is qualitatively similar to Fig. 4A, other than the endpoints, which now reflect the wild-type excess of PrE over Epi cells. Early and late pulses have little effect, whereas intermediate pulses are most effective in changing fate.

Our model derives the internal FGF from the current state of the cells and implicitly assumes that the internal FGF instantaneously reflects the current state. The effects of making FGF a dynamic variable that reflects the state of the cell with a lag are quite minor (Fig. S5A). The cell decisions take place asynchronously and the sensitivity to FGF is at late times, as shown in Fig. 5A, so the system tolerates some lag in FGF production because of the topography.

To model a competence period, we allow the second parameter of our potential *K*_2_, which we have so far held fixed, to linearly decrease in time and transition through the value where the ICM state bifurcates away. (The molecular control of this decision is unknown, but the phenomenon is very clear.) We map the computation time linearly between E2.25 and E4.75, corresponding to the point when most cells in the model are close to *x*=±1, i.e. Epi or PrE. The rate that *K*_2_ decreases is fitted to the disappearance of ICM in Fig. 5B.

The first experiments to define the competence window for the response to FGF involved culturing the blastocyst in control media and then adding FGF4 or inhibiting the MAPK pathway (arguably the converse) (Yamanaka et al., 2010). The specification process was captured in more detail by Saiz et al. (2016) by imposing thresholds on GATA6 and NANOG to define three states (which required us in the model to define a range of *y* above 0.5 corresponding to ICM). The transition from ICM to PrE or Epi occurred in an asynchronous fashion that we fit in Fig. 5B. Dynamic noise leads to the error bars in Fig. 5D.

Experiments by Bessonnard et al. (2017) divided the critical period for response to FGF4 into 2 h intervals and suggested the temporal sensitivity that we predict in Fig. 4. However, their protocol extended the exposure times in 2 h increments, rather than keeping the duration fixed and varying the application time. The maximal sensitivity of the cells to FGF4 application fell between E3.5 and E3.75, whereas FGF inhibition had it biggest effect between E3.5 and E4.5. As the authors note, this could be explained by a time lag in the production of FGF given the cellular state.

Finally, recent experiments have shown very directly that the blastocyst makes a population-level decision to control the ratio of Epi and PrE cells (Saiz et al., 2020). The authors combined *Gata6*-null cells, which are unable to specify PrE with wild type in different proportions, and observed the fate of the wild-type cells. When the fraction of wild type was below 60% (the wild-type fraction of PrE), essentially all the wild-type cells converted to PrE cells, indicating that internal population-level controls are very strong. We model the Gata6^−/−^ mutants by changing the initial conditions so they are already in the Epi state, and find essentially the same linear dependence of PrE fraction on wild type between 0 and 60-80% wild type (Fig. 5E).

## DISCUSSION

### A geometrical understanding of cell fate decisions

Previous models of the cell fate in the ICM have usually focused on the NANOG-GATA6-FGF/ERK regulatory network. The connections between these components are still not fully clarified and different models assume different kinds of regulation. It is difficult to determine precisely the parameters in such nonlinear models. Geometrical models bypass this difficulty by modeling only the cell fate. Furthermore, when any gene-centric model makes a decision between three fates, it must follow one of the bifurcations delimited by mathematics, so those bifurcations should be the subject of modeling from the start (the parameters in more-elaborate models must collapse near the transition into those describing the bifurcation, and how they combine is problem dependent and was successfully negotiated by Corson and Siggia, 2017).

When extended to the entire embryo by means of an averaged internally generated FGF, the geometric model had the unexpected feature of being rather insensitive to how the FGF is derived from the underlying state of the cells in the landscape. Either an instantaneous function or a lag time of up to 1 day gave similar results. The FGF averages over all Epi-like cells and the movement of that population in the topography imposes a window on when the FGF can influence fate. At early times, the cells are still confined in the valley leading from the ICM state; at late times, the cells are captured by either the Epi or PrE fixed point.

Multiple genes define a lineage, and theory predicts that the dynamics of these genes are ultimately determined by the variables describing the bifurcation (as can be shown by taking a gene-centric model and extracting the bifurcation). However, this determination can vary quantitatively as genes or combinations will relax at variable rates to the geometric description, so an underlying model to define the tendencies is essential for analyzing data. From the data in Simon et al. (2020), the fate of individual cells given a 2 h sample of the ERK is not very predictive of the ultimate fate, but the embryo as a whole achieves an accurate partitioning of cells into Epi and PrE, evidently by averaging many imprecise decisions.

From a list of the allowed bifurcations, dual cusp and flip in the present context, it becomes easier to design experiments that distinguish them. Although we believe most labs would favor the flip when it is defined, no experiment explicitly rules out the dual cusp, to our knowledge. Time-dependent perturbations are an ideal way to distinguish models, and a geometric visualization makes their outcomes transparent, as illustrated by the qualitatively different responses of the dual cusp and flip to a pulse of FGF shown in Fig. 4D,E. Similarly, Fig. 3 shows that if a pulse of FGF is applied before lineage commitment, the dual cusp requires a transition back through the ICM, whereas the flip does not. Using a model, quantitative experiments that use a modest dose of morphogen to perturb outcomes are more informative than treatments that give all or none responses because they probe the boundaries between states.

Previous models of the ICM transition illustrate the difficulties incurred by being too specific. The model of Tosenberger et al. (2017) has four dynamic equations with 24 parameters (plus additional equations for the FGF); however, near the point of ICM instability, their model is equivalent to a dual cusp (see De Mot et al., 2016).

We have two equations with two free landscape parameters (one time dependent) in need of fitting (plus additional constants for the FGF). The model in Saiz et al. (2020) assumed only bi-stability between PrE and Epi in one dimension, but had to initialize the cells at the dual positive (NANOG+GATA6) saddle point, which is difficult to imagine, although their data are consistent with the two-dimensional flip geometry. The model of Stanoev et al. (2021) proposed an inverted pitchfork bifurcation in which the ICM disappears to produce two new fixed points corresponding to Epi and PrE. The authors fixed the cells to a 2D lattice and assumed variable range FGF signaling, so the spatial geometry was tied to the cell fates, in a manner similar to Turing systems with boundaries or Notch-Delta repression. The data from Simon et al. (2020) show considerable cell movement and PrE markers that appear before spatial segregation, so we consider the conclusions of Stanoev et al. (2021) to be too contingent on assumptions.

We have emphasized the flip topography for its logical consistency (i.e. we avoid the parameter tuning inherent in the dual cusp or pitchfork). The flip, which is a global bifurcation, is ideally suited to transitions where the embryo needs to control the population ratio via morphogen feedback. The transition involves a reconnection of the orbit leaving the progenitor from one terminal state with the other. In our case, the progenitor destabilizes; however, in other situations, it remains an attractor and cells exit via fluctuations (Sáez et al., 2022b). Signals have a finite time to act on the orbit and effect the flip.

### Model assumptions and their validity

A clear difference between our model and previous models in the literature is the role that spatial interactions play. Most previous models have considered the FGF signaling to mediate very localized spatial interactions, although a recent study suggests longer-range signaling (Fischer et al., 2023). A recent study measured the spatial range of FGF signaling in a mouse embryonic stem cell system by creating a cell line with a FGF4 transcriptional reporter (Raina et al., 2021). Raina et al. estimated that the spatial range of FGF signaling was two spatial neighbors.

Although it may be true that FGF signaling *in vivo* is also somewhat localized, we do not find evidence of spatial correlations at the level of the ERK activity, perhaps due to cell movement. We find that cell movement scrambles neighbors every 30 min, which is quick compared with the typical timescale of the decision (1 day as in Fig. 5B). In our model, we assume a single homogeneous but time-dependent FGF concentration. Current data do not prompt us to contemplate anything more complex.

However, Kang et al. (2013) infers the need for localized FGF interactions working with blastocysts with *Fgf4* knocked out. They found that exogenous FGF applied during the growth from 32 to 100 cells produced embryos that were either entirely Epi or entirely PrE at the end, but passed through an earlier stage of mixed NANOG-GATA6 expression. Exogenous FGF did not rescue the knockout, yet the FGF level was not extreme, as evidenced by the dual outcomes (all Epi or all PrE). Perhaps FGF needs to be finely tuned to achieve a balanced outcome in the absence of feedback. It would be interesting to repeat this experiment with a live ERK reporter and track cell motility to see whether the dynamics replicates Simon et al. (2020). It may also point to a backup mechanism for enforcing homogeneous fates based on integrin- or cadherin-mediated contacts.

We have assumed that one parameter in the landscape controls the bifurcation of the ICM and a separate parameter receives FGF input. In reality, the two are probably mixed, but current experiments do not allow us to disentangle the mixing. The model relating the internally generated FGF to the cell state, is phenomenological. We have assumed the readout of cell state is instantaneous, but time lags are plausible.

Cell division and the correlation between the fates of the daughters plays no role in our model. If a cell divides in a diverging region of the flow plane, the correlation between the daughters would tend to make a final Epi to PrE ratio a bit noisier than in the absence of correlation, but that statistic does not yet figure in the model fits. The effect could be mimicked by adjusting our noise source.

Our competence period of the cells for the FGF signal is set by a separate event that bifurcates the ICM at a specified time. Such signals are known in other contexts. For example, in germ layer specification in zebrafish, a recent study reported that Nodal signaling set the competence window for FGF to regulate fate (Economou et al., 2022). Our assumption is consistent with previous studies that show the cells becoming competent to FGF signaling at a specified time post-fertilization (Nissen et al., 2017). How the embryo keeps time or counts cells is an open and interesting question.

In addition to timing, experiments where the levels of FGF and inhibitors are titrated down towards a threshold would define a presumably sigmoid response to FGF (and, by comparison, the level for what is produced internally). Partial penetrance is a way to infer the boundaries between fates, not the hindrance that it would be in genetic screens. Experiments that control timing and deal with partial penetrance require a model, as the outcomes are quantitative not binary. Ultimately, our model seeks to encourage and help interpret future experiments, such as we propose in Fig. 4, to study this paradigmatic transition in greater detail.

## MATERIALS AND METHODS

### Spatial correlations

The correlations in Fig. 1 are calculated for the data of Simon et al. (2020). The spatial correlations are calculated using the 2 h movies (imaged at 5 min intervals). We remove all cells that have the TE fate (manually labeled). We estimate the average time taken for one neighbor of a cell to change by taking every embryo that has at least 10 cells, calculating the Voronoi neighbors for each cell and calculating the average time it takes for one unique Voronoi neighbor to change (i.e. if the same neighbor is lost and gained in the 2 h time period, we count it only once). This turns out to be around 30 min in the data. We thus measure spatial correlations from data separated by 30 min (i.e. four data points from one 2 h movie). We measure spatial correlations using the Moran's I index. We use a weight matrix *w*_*ij*_, which is row-standardized so 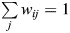. The statistic is then given by:
(1)

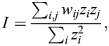
where *z*_*i*_ is the data that are centered around the mean. Given a physical distance *d*_*ij*_ between two cells indexed by *i* and *j*, we define 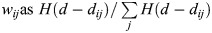, where *H* is the Heaviside theta function (which is 1 for distances below some fixed distance *d*=20 μm and 0 otherwise). The value of *d*=20 μm is approximately the mean distance between Voronoi neighbors in the data. We calculate this statistic for snapshots of embryos.

The pair distances are taken from the starting time point and the last time point in the 2 h movies. The distance *d* is calculated by averaging over nearest neighbor distances at the starting time point. To calculate the number of neighbor changes reported as *N*_*s*_ for one embryo, we first calculate the number of unique changes in the adjacency matrix obtained from the Voronoi construction over the 2 h time period. A single element of the adjacency matrix may change several times over the 2 h time period and it is counted only once. Each change corresponds to a change in the neighbor of one cell (as the adjacency matrix is symmetric, changes always come in pairs and correspond to changes in the neighbors of two cells). Hence, the total unique changes in the adjacency matrix divided by the number of cells gives the average number of neighbor changes per cell in the embryo. When this is done for all embryos with at least 10 ICM-derived cells, it gives an average of one neighbor change per cell every 30 min, as reported above (see supplementary Materials and Methods for further details).

### Equations for potentials

The potentials for the dual cusp and heteroclinic flip were modified from Sáez et al. (2022b). For the dual cusp:
(2)

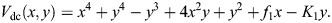
For the heteroclinic flip:
(3)

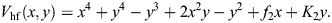
For Fig. 2, we used *K*_1_=0.15, *K*_2_=1.4, *f*_1_=0, *f*_2_=0. The potentials in Fig. 2 are drawn only to emphasize the topology. The flow lines and bifurcation diagram are drawn using the above equations.

For a given potential *V*(*x*, *y*), the flow is modeled as:
(4)

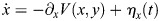


and
(5)

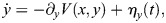
where *η*_*x*_(*t*) and *η*_*y*_(*t*) are independent noise terms, such that 〈*η*_*x*_(*t*)*η*_*x*_(*t*^′^)〉=*σ*^2^*δ*(*t*−*t*^′^) and 〈*η*_*y*_(*t*)*η*_*y*_(*t*^′^)〉=*σ*^2^*δ*(*t*−*t*^′^). We choose *σ*=0.05. The initial condition is chosen as a small Gaussian distribution (s.d.=0.05) around the fixed point corresponding to the ICM state for zero FGF concentration and *K*_1_≈1.45 (*x*=0, *y*=0.8) for the flip, and *K*_2_≈0 (*x*=0, *y*=0) for the dual cusp.

For Fig. 3, we chose *K*_1_=− 0.05 and *K*_2_=1.55. The initial conditions for the dual cusp are drawn from a normal distribution with means of 0.3 and − 0.2 in *x*, *y*, respectively, which is towards the Epi state. For the heteroclinic flip, the initial conditions are drawn from a normal distribution with means of 0.1 and 0, respectively, towards the Epi state. In both cases, *σ*=0.05 and external FGF *f*_1_, *f*_2_=0.2. Streamlines are shown at the bifurcation of the ICM state.

For Fig. 4, we chose *K*_1_=− 0.05 and *K*_2_=1.55, *σ*=0.05. The external FGF is given as a pulse of magnitude 0.2 and a duration of 0.5. Identical realizations of a slightly larger noise (*σ*=0.1) are used in Fig. 4A-C for demonstrative purposes. Streamlines are shown at the bifurcation of the ICM state. The plots in Fig. 4D,E are averages over 2500 runs of single cells.

### Dynamical equations for full model

For the full model, cells interact via the common FGF and follow the flip landscape. Each one of them has the equation:
(6)

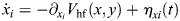


or
(7)

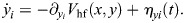


The noise terms are defined as before, with each cell seeing a different realization sampled with an identical standard deviation (*σ*) in both directions. The time of cell fate specification is normalized to be between 0 and 10, with a scale factor to embryonic time *τ*. Time *t*=10 is assumed to be the end of the competence period, when *f*_1,2_ revert to 0. The bifurcation of the ICM state is done globally for each of the cells by making *K*_2_=*m*_0_+*m*_1_*t* with *m*_0_=1.45 and *m*_1_=0.022, where *t* is time. The bifurcation happens around *t*≈2.5. The parameter *m*_1_ controls how rapidly cells leave the vestige of the ICM state. (In Sáez et al. (2022b), the analogue of the ICM state never lost stability and the cells exited due to the noise.) As data are available in Fig. 5, this allows us to fix our map with *t*=0 equivalent to E2.25 and *t*=10 equivalent to E4.75 and a unit difference corresponding to 0.25 days.

The FGF concentration is calculated using
(8)

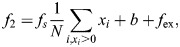
where the first contribution is only from those *x*_*i*_ that are positive and represents the feedback from the Epi cells. The second is an initial bias towards the Epi state and the third is the effect of external FGF. We set *b*=−0.004, *f*_*s*_=0.1 and *f*_ex_=0.2 for FGF overexpression and *f*_ex_=−0.3 for the inhibitor. In principle, the inhibitor would affect the *f*_*s*_ term, which we have not modeled. *N* is the number of cells (taken to be 50).

To simulate a lag in the signaling, we change the internal FGF into a dynamic variable and the feedback from the Epi cells has an associated timescale *τ* (see supplementary Materials and Methods).

### Comparison with experiments

The coefficients in the polynomials in Eqns 2 and 3, other than those denoted symbolically, are not fitting parameters. Other choices that differ modestly from those shown could be absorbed by a change in variables. As we associate fates with fixed points and the topology of the flows connecting them will not change, the variable change is of no consequence.

We have seven important parameters in our model: three to define the FGF feedback in Eqn 8, three for the timescales (the slope and origin of *K*_2_, and the mapping of embryonic to computational time) and one to delimit ICM for the terminal fates (Fig. 5A). Of these seven, three are unavoidable definitions of units (of FGF concentration and time). The threshold to delimit ICM is unavoidable. The bias in FGF is needed to capture the asymmetry in PrE and Epi. The form of *K*_2_ is required to fit the competence period and asynchronous loss of ICM character. Apart from these seven, the noise in the equations, *σ*, and the variability in timing of the FGF signal are less important. The initial conditions around the ICM state are similarly not very important.

We set the timescales by comparing with Fig. 5B. We set the scale of the internal FGF by comparing with Fig. 5E. The scale of the external FGF is set by comparing with Fig. 5D. The media is assumed to be switched exactly at E3.75 but it is possible to assume a slight variability in time that could explain the discrepancies from data. The data are taken from Saiz et al. (2016).

For comparing with the experiments shown in Fig. 5E, a random proportion of cells are assumed to be in the Epi state (the Gata6 mutants). The rest are assumed to start at the ICM state. We then run our simulations several times (1000 runs) and bracket our results the same way as Saiz et al. (2020) by the number of cells that are mutants versus wild type. The data are taken from figure 1(S2)-L in Saiz et al. (2020).

## Supplementary Material



10.1242/develop.202467_sup1 Supplementary information
