# Supplementary material for: A geometrical model of cell fate specification in the mouse blastocyst
Source: Development. 2024 Apr 22;151(8):dev202467. doi: 10.1242/dev.202467 (PMC11112346; doi:10.1242/dev.202467)
Supplement: Supplementary information [file develop-151-202467-s1.pdf]

Supplementary Materials and Methods

Figure S1 has the change in distances between pairs of cells over the 2 hours period. The procedure for calculating d and N_s is described in the main text. Figure S2 shows how pair contacts change with time.

The ERK correlation shown in Figure S3 are calculated by taking a random cell in each embryo, and finding the correlation between the time series of the cell and that of those neighbours that remain neighbours over the 2 hour time period. We only take differences in ERK C:N level from one time to the next while calculating the correlations. The histogram of correlations has mean near zero.

The lineage statistics shown in Figure S4 are calculated from the 12 hr movies (imaged at 15 minute intervals). We take all cells which have undergone a mitotic division, and plot their mean ERK C:N Ratio as well as the absolute difference between the ERK C:N Ratio for the two sisters for a 2 hour time period (8 data points).

The lag is modeled with the by modifying Equation 8 in the main text as follows.

$$f_2 = f_{\text{int}} + b + f_{\text{ex}}. \tag{1}$$

f_{int} is now a dynamical variable and has the following equation.

$$\dot{f}_{\text{int}} = \frac{1}{\tau}\left(-f_{\text{int}} + \frac{f_s}{N}\sum_{i,x_i>0} x_i\right), \tag{2}$$

with τ setting the time scale of the internal FGF response. These equations along with the ones in the main text are used for Figure S5. All other parameters are kept the same as in the main text.

Development • Supplementary information

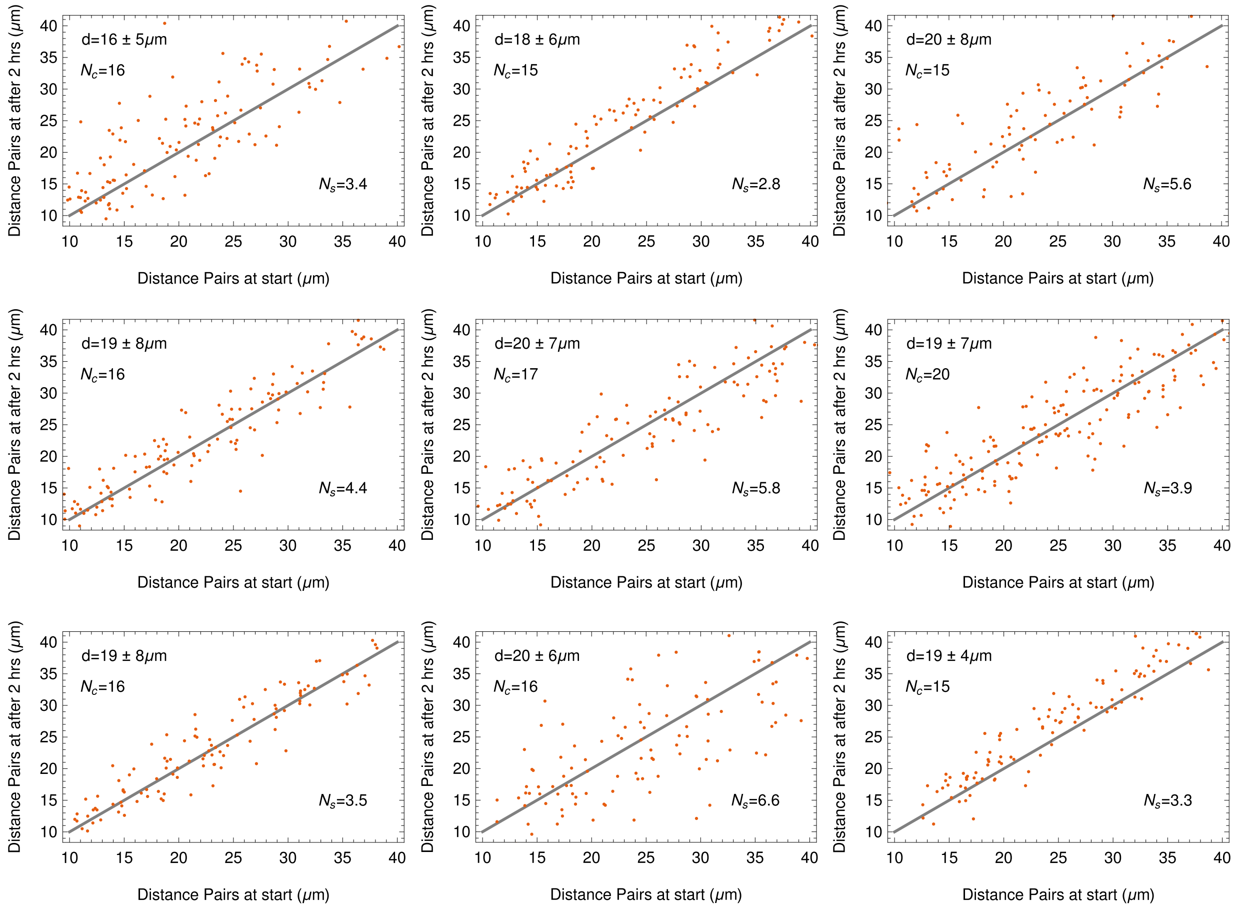

Fig. S1. A plot of the change in distance between pairs of cells (excluding TE cells) shown for nine embryos over a period of 2 hours. We only report distances for cells whose position is reported at both time points (i.e. we ignore cells that divide or are lost in tracking, which occasionally happens in this time period). The gray line is drawn as a guide and has slope 1. N_c is the number of cells (excluding TE cells), d is the average pairwise distance between Voronoi neighbours at the starting time. Cell movement leads to change in Voronoi contacts. The total number of unique pair contact changes divided by N_c gives the average number of neighbor changes per cell N_s in 2 hours which is reported for each embryo (see Figure S2).

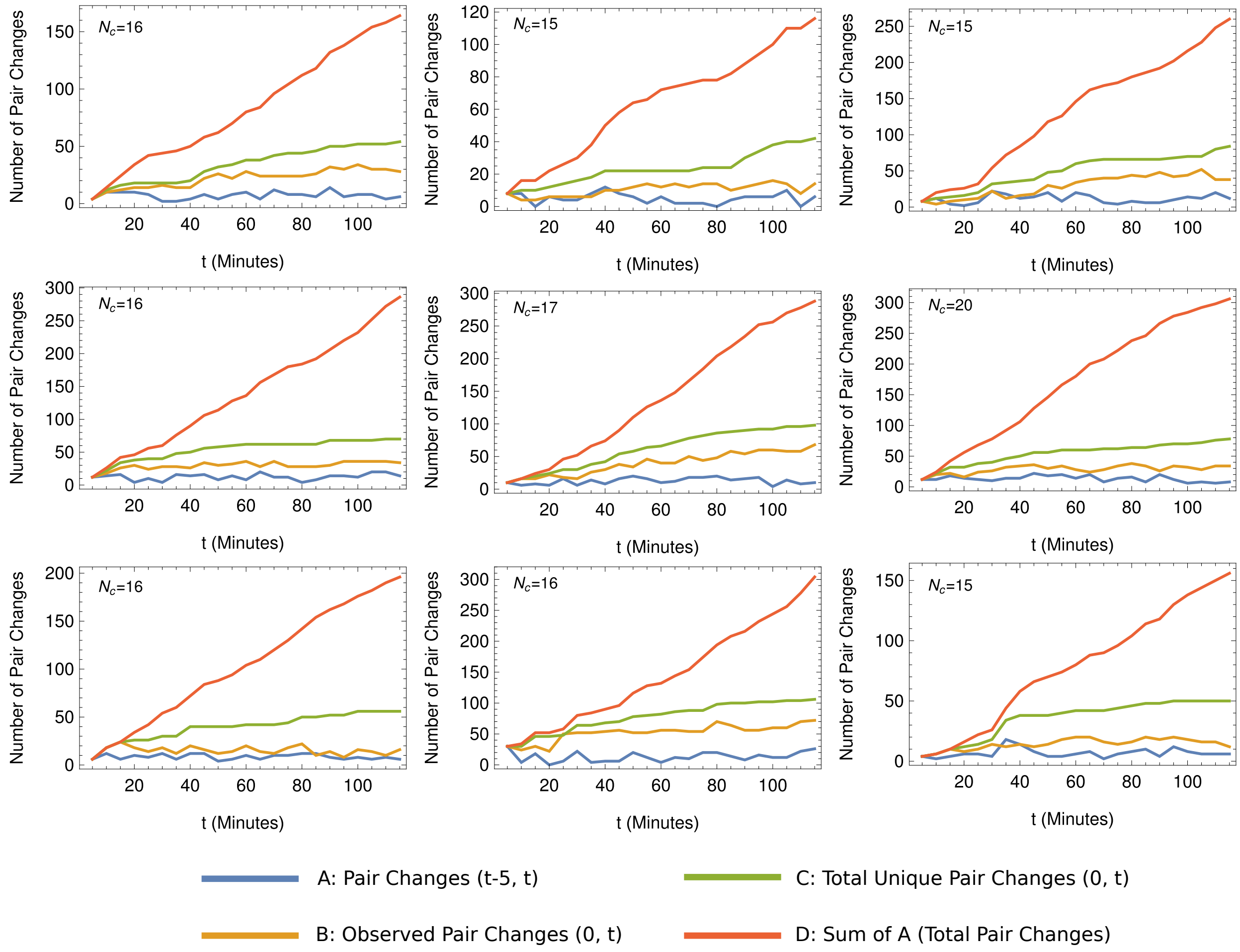

Fig. S2. A plot of the change in pair contacts as defined by the Voronoi construction. The blue curve A shows the change in pair contacts every 5 minutes. The yellow curve compares the adjacency matrix at time t and start time. The green curve is calculated by summing the difference between adjacency matrices at subsequent time points, but only counting unique changes to the matrix. The red curve is simply the sum of A over time. The cells often move back and forth so pair contacts are lost and then gained at a future time point. The yellow curve does not count such events. The red curve counts all such events. The green curve counts such changes only once and thus avoid overcounting. Rarely, cells divide or are lost in the tracking. For the purposes of this calculation, whenever calculating the change in the adjacency matrix between two time points, we only use cells whose positions are reported for both time points. N_c is the number of cells (excluding TE cells) common to the first and last time point. The value of the green curve at the last time point is used to calculate N_s reported in Figure S1.

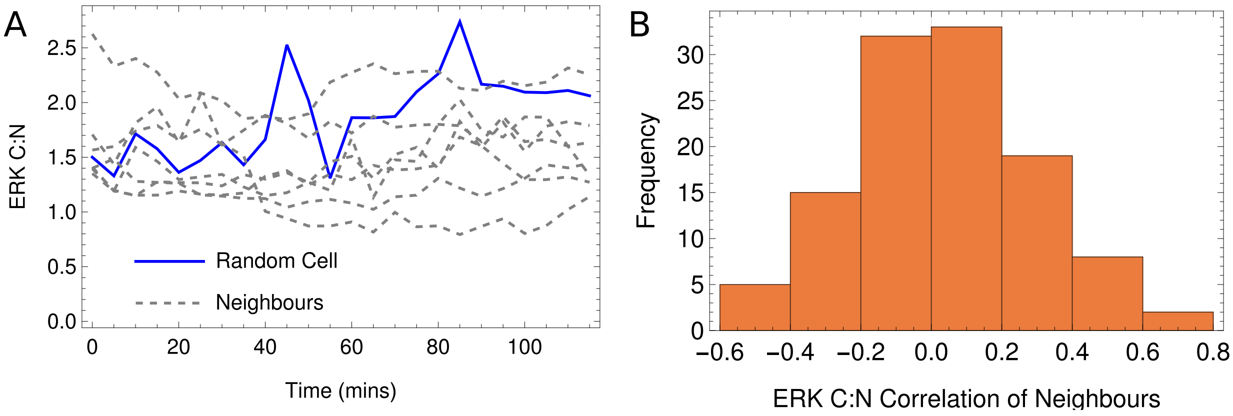

Fig. S3. (A) Example of the ERK C:N of a randomly chosen cell in one embryo alongside that of its Voronoi neighbors that remain in contact for the 2 hour period to show the extent of the fluctuations. (B) The correlation of the time series of ERK C:N with that of its Voronoi neighbours. We take the difference of ERK C:N at successive time points to calculate the correlations between a cell and its neighbors. This checks if the fluctuations in the ERK C:N of a cell and its neighbor are correlated. These correlations are calculated for a randomly chosen cell from each embryo and a total of 114 values are used to draw the histogram of correlations which has mean 0.04.

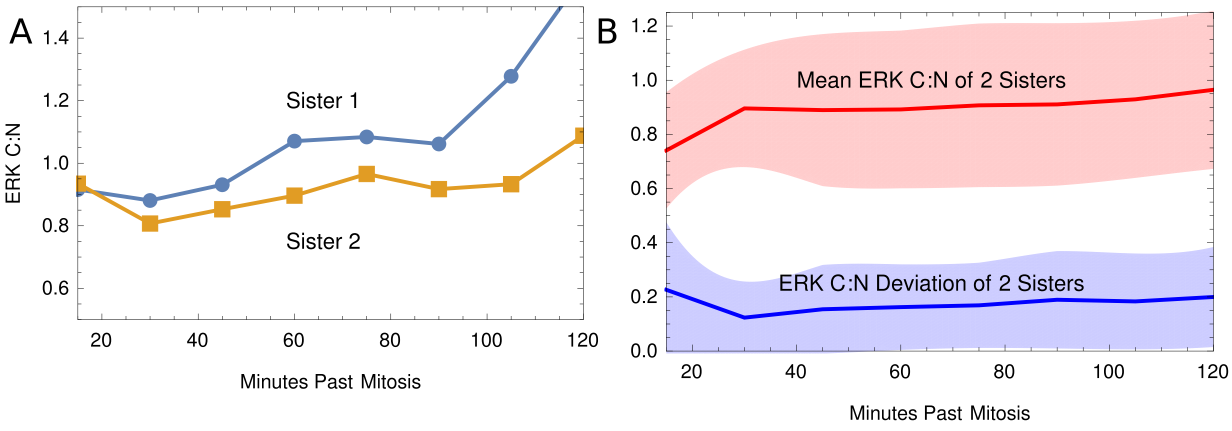

Fig. S4. (A) An example of the ERK C:N trajectory of 2 sister cells after mitosis. (B) The mean ERK C:N levels of two sister cells after mitosis plotted alongside the absolute deviation of ERK. The deviations are substantially smaller than the mean reflecting correlated lineages. A total of 111 cells are used for the plot with 8 data points each corresponding to a 2 hour time period. Shaded regions are one standard deviation.

Development • Supplementary information

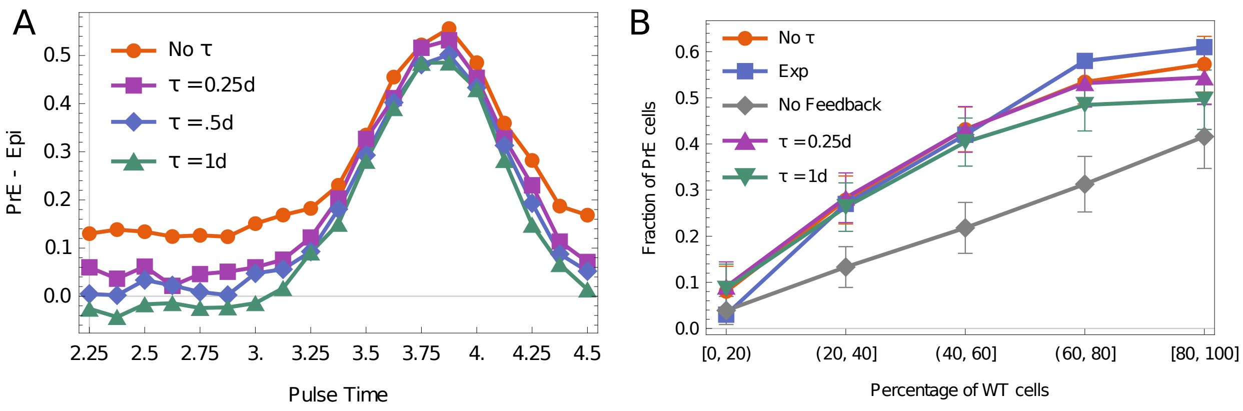

Fig. S5. We show the change in predictions if a lag is allowed in FGF signaling with time scale τ (in days). We assume the external FGF is instantaneous but the internal FGF has a time scale. Wild-type levels change because we only change τ and keep all other parameters constant. Note $\tau \to 0$ is the limit of instantaneous FGF response giving the model in the main text. (A) The response to a pulse of FGF signaling for different τ. The red curve is our model in the main text. Lags tend to shift the curve down but don't change the shape of the curve drastically. (B) Comparison of the results of our model from the data in Saiz et al. (2020) where Gata6$^{-/-}$ cells were combined with wild-type cells in different proportions (see Figure 5E in main text). The results with the same set of parameters but with an added lag are shown in purple and green for two different τ values. The gray curve shows the case with no internal feedback for comparison.

Development • Supplementary information
