# Supplementary material for: A geometrical model of cell fate specification in the mouse blastocyst
Source: Development. 2024 Apr 22;151(8):dev202467. doi: 10.1242/dev.202467 (PMC11112346; doi:10.1242/dev.202467)
Supplement: Supplementary file 1 [file develop-151-202467_review_history.pdf]

A geometrical model of cell fate specification in the mouse blastocyst

Archishman Raju and Eric D. Siggia
DOI: 10.1242/dev.202467

Editor: Paul Francois

Review timeline
Original submission: 23 October 2023
Editorial decision: 19 December 2023
First revision received: 9 February 2024
Accepted: 20 March 2024

Original submission

First decision letter

MS ID#: DEVELOP/2023/202467

MS TITLE: A geometrical model of cell fate specification in the mouse blastocyst

AUTHORS: Archishman Raju and Eric Siggia

Thanks for submitting your work to Development !The referees' comments are appended below, or you can access them online: please go to BenchPress and click on the 'Manuscripts with Decisions' queue in the Author Area.

As you will see, the referees express considerable interest in your work, but they recommend a revision of your manuscript before we can consider publication. I will be more than happy to receive a revised version of the manuscript addressing their points. Your revised paper will be re-reviewed by one or more of the original referees, and acceptance of your manuscript will depend on your addressing satisfactorily the reviewers' major concerns.

Please also note that Development will normally permit only one round of major revision. If it would be helpful, you are welcome to contact us to discuss your revision in greater detail. Please send us a point-by-point response indicating your plans for addressing the referees' comments, and we will look over this and provide further guidance.

Please attend to all of the reviewers' comments and ensure that you clearly highlight all changes made in the revised manuscript. Please avoid using 'Tracked changes' in Word files as these are lost in PDF conversion. I should be grateful if you would also provide a point-by-point response detailing how you have dealt with the points raised by the reviewers in the 'Response to Reviewers' box. If you do not agree with any of their criticisms or suggestions please explain clearly why this is so.

Reviewer 1

Advance summary and potential significance to field

In this paper the authors study the generation of epiblast and primitive endoderm from the inner cell mass (ICM). They apply bifurcation theories in order to investigate the decisions in the

© 2024. Published by The Company of Biologists under the terms of the Creative Commons Attribution License (https://creativecommons.org/licenses/by/4.0/).

development. Indeed, they find that a fairly complex phenomenon, a heteroclinic flip bifurcation, is the likely transition that occurs for the single cells. The paper provides a simple and elegant way to elucidate the cell fate segregation in ICM development with the geometrical model.

The paper is quite well written and I find the application of bifurcation analyses interesting and appealing in the context of cell fate specification in the mouse blastocyst. I believe such analysis has not been performed before. On the basis of this I am positive against publication of the paper.

Comments for the author

I however have the following comments.

I find the comparison of results in Fig. 2 very appealing where two possible geometries (the dual cusp and the heteroclinic flip) are proposed.

I have one concern about the part predicting cell proportions with different FGF4 perturbations. At the beginning of the paper, the authors justify the assumption with data analysis. They conclude that the whole ICM tissue senses FGF4 signaling (or cell fate decision based on the FGF4 signaling) uniformly. It appears to me to me a somewhat strong assumption. (In this connection, there is quite interestingly a paper proposing an opposite opinion about it:
https://www.sciencedirect.com/science/article/pii/S2589004223021831?via%3Dihub)

In relation to the landscape structure and the relationship between FGF4 and cell proportions, the authors write "model FGF as proportional to the sum of the x coordinates of all cells". This assumption is justified only if the final steady states are analyzed.

However, I am a little confused about how they argue that FGF concentration is proportional to the sum of Epi cells during the developing time if FGF4 is not quickly degraded.
In the methods, the authors mention that they are comparing the time scale to the experiments (Line 356), which I think is not obvious. Do they use the same one for all the experiments? What is the value like as compared to the whole simulated time?

When FGF4 changes, the landscape will change, and the cell fate will change, in turn, the FGF4 will change. Thus if FGF4 does not tightly follow the Epi proportion (fast time scale), and the analysis based on the replacement of FGF4 with Epi cell proportion fit the dynamics in the experiment is not convincing. It also means the authors probably need to introduce a separate FGF4 viable to explore if the model is capable of explaining the cell proportions under different conditions. Or the external FGF4 effects directly overwrite the effects of internal FGF4 as they write in equation (8).
I hope that the authors can resolve these issues or make it crystal clear within their modelling framework.

In conclusion, I find this an appealing paper. Above I have raised some issues which should be elucidated after which the paper could be considered for publication in Development.

Reviewer 2

Advance summary and potential significance to field

This paper continues in the productive and powerful line of work on geometric modeling of cell fate dynamics. Going beyond the infrastructure laid out in prior publications this work very powerfully embodies the ways in which we can make the most of topologically minded thinking in modeling cell fate dynamics. It is my view that the authors make many biologically motivated and data-driven assumptions/simplifications that constrain and simplify the possible geometries.
These choices are essential to the modeling endevour and done beautifully. For me this paper best embodies how we should model. Surely it also provides insight into the particular biological problem of interest, early mouse development.

Comments for the author

© 2024. Published by The Company of Biologists under the terms of the Creative Commons Attribution License (https://creativecommons.org/licenses/by/4.0/).

Is the 25 minute estimate the change in vornoi neighbors of every cell? Or is there on average one change in the adjacency matrix every 25 minutes across the whole embryo? Can the data for neighbor exchanges be presented? I believe this is crucial.

From fig1D is certainly seems like cell motion is correlated amongst cells that are closer to each other. Red and green trajectories look highly correlated. Same with cyan and magenta. This would suggest that neighbors dont change as if the system was really “well mixed”.

A more careful time series analysis would be useful. Within the 25 minute window are the erk fluctuations between cells that are in contact (in the vornoi sense)
correlated? Meaning, what is the correlation coeffieicnet between neighboring cells as calculated through a time series analysis within a 25 minute window? This would shed light on whether space matters.

Can the Moran statistic please be defined in the manuscript itself? Surely inline equations should be permitted, and used, in such quantitative studies.

In figure 1b the y axis is the mean of neighbors. This might obscure spatial correlations that might exist between certain pairs. I realize that this is larger probability distribution, but what does the scatter of c:n look like of a cell and its neighbors? Is it bimodal? Some, or few, neighbors show significant correlation and most dont? That would suggest that the mean isn’t the right statistics to use.

With regards to the lineage analysis. Could the authors show us an example of what the time series looks like right after a division in sister cells? Could the individual trajectories also be shown instead of the 1sd cloud around the mean?
Could a correlation between sister times series be performed after the hangover from the mitosis is over — which seems to last on average around 20-30 minutes? It just struck me as to whether the “mean erk” in the lineage analysis is of the c:n ratio itself? Can the authors clarify whether for this lineage analysis they are summing averaging c and n, or is the mean referring to the ratio?

As a whole, I would like to say that the overall philosophy of making the ansatz that for some reason thinking of the fgf as not spatially resolved, but time-dependent, is valuable. Its accuracy can be judged based on the accuracy of the ensuing geometric models ability to fit the data and predict novel aspects. That all being said, I think the time series analysis perform in figure 1 falls short.
More can be done, even if it only furthers the deductions made about the irrelevance of space in the dynamics of differentiation. One important timescale that I believe the authors know, and should either report or estimate, is some kinetic timescale of signaling itself. The 25 minute number must be compared to something else. The something else ought to naively be some timescale associated with signaling itself, which surely is known from other fgf signaling studies. If 25 minutes is slow/large compared to this intrinsic timescale then space matters potentially. If 25 minutes of the neighbor exchange timescale is fast/small compared to the intrinsic signaling timescale then one can neglect space since the cell so to speak samples multiple signaling sources within one signaling half life.

The beginning of 2.2 can have a little bit more background. Some words associated with saying that there is a categorization of 3 attractors in 2 dimensions and that this has been leveraged by the authors to model differentiation. Just even a little context brings the readers into the world view that this current paper belongs to. In addition, I would ask the authors to explain the reasons that thinking about locality, or lack therefore, and the dimensionality of decision, is valuable as far as the biology goes. To those of us who know local and global, the circle encompassing the fixed points means something. I can imagine to the less mathematically informed reader the word local/global, or the circle construction doesn’t help. Same goes for one vs two dimensional. Can these concepts, which are so immensely powerful/universal/generic be interpreted biologically for that kind of audience? If these ideas are to permeate then the leaders in the field, I believe, must provide interpretaion and context!

For section 2.3: I really wish the authors would pose the incredible value of this section in a manner that could be read and understood by a biologist. The real key fact, in my limited view, is that it is

© 2024. Published by The Company of Biologists under the terms of the Creative Commons Attribution License (https://creativecommons.org/licenses/by/4.0/).

generically observed that the responses of marker genes to signaling perturbations can look entirely non trivial. In particular, the response of the system can be non-monotonic in time. This is a very counter intuitive observation since the loose intuition of your average dev bio person is that commitment increases in time and thus response to signals should always be reducing as the ball falls into a well. This very nice example that the authors show demonstrate that for very good, in fact even more generic reasons, you should expect a non monotonic response functions. In this sense, of course, the words the authors use are entirely spot on. But I would be so much happier if they spend a little real estate making all these connections and analogies to help it and for the biologically oriented reader. Otherwise, sadly the work of this qualitatively minded community will only be cited by others within, and not without.

This sentence in the discussion “.The flip, being a global bifurcation, is ideally suited to transitions where the embryo needs to control the population ratio via morphogen feedbacks “Is deep. More should be said. It is deeply related to the circle construction at the start.

First revision

Author response to reviewers' comments

Reply to Referees:

Reviewer 1
1. *I have one concern about the part predicting cell proportions with different FGF4 perturbations. At the beginning of the paper, the authors justify the assumption with data analysis. They conclude that the whole ICM tissue senses FGF4 signaling (or cell fate decision based on the FGF4 signaling) uniformly. It appears to me to me a somewhat strong assumption. (In this connection, there is quite interestingly a paper proposing an opposite opinion about it: https://www.sciencedirect.com/science/article/pii/S2589004223021831?via %3Dihub)*

We thank the referee for this reference, which just appeared in Nov 2023, so after our paper was submitted. They do not reference the papers of Simon etal and Pokrass et al Dev Cell 2020 that were the basis of our analysis. They look only at fixed samples and classify cells based on the expression of Nanog and Gata6, they do not have movies to trace the movement of individual cells. There is obviously a spatial clustering of similar types for late blastulas since PrE and Epi cells have segregated. For earlier blastocysts they reject a model of nearest neighbor FGF signaling and conclude:

> “The spatial patterns of NANOG- and GATA6-expressing cells in early and mid blastocysts are consistent with a mechanism that integrates intercellular signaling from the whole ICM without cell division”

So we interpret this as compatible with our conclusions, but emphasize that we trace cell movements and do not just look at spatial correlations in fate. We have modified Fig. 1 to better display the very minimal correlations in fate we find, plus the cell movements. Additional data in a new supplement Figs S1-3.

We did cite two papers that proposed localized FGF signaling, Refs 16, 24. Only the second of these is experimental, but with stem cells in vitro, so we consider our evidence from movies of blastocysts better. We have now also cited this reference in the discussion.

2. *However, I am a little confused about how they argue that FGF concentration is proportional to the sum of Epi cells during the developing time if FGF4 is not quickly degraded. In the methods, the authors mention that they are comparing the time scale to the experiments (Line 356), which I think is not obvious. Do they use the same one for all the experiments? What is the value like as compared to the whole simulated time?*

This is a good question, and we have now modified the model to include an explicit lifetime on the

© 2024. Published by The Company of Biologists under the terms of the Creative Commons Attribution License (https://creativecommons.org/licenses/by/4.0/).

FGF, so that it integrates up the instantaneous contribution from the Epi cells proportional to each value of x. The results are shown Figure S5A and the subject of a new paragraph in Sec 2.4 and a Discussion remark. There is little change; for the reason see reply to item #3 below.

3. When FGF4 changes, the landscape will change, and the cell fate will change, in turn, the FGF4 will change. Thus if FGF4 does not tightly follow the Epi proportion (fast time scale), and the analysis based on the replacement of FGF4 with Epi cell proportion fit the dynamics in the experiment is not convincing. It also means the authors probably need to introduce a separate FGF4 viable to explore if the model is capable of explaining the cell proportions under different conditions. Or the external FGF4 effects directly overwrite the effects of internal FGF4 as they write in equation (8).

Referee makes a good point that we have now amplified in Sec 2.4 when we introduce our model for the internally generated FGF. We ran with FGF an instantaneous function of the cell states, which we now correct to include a lifetime, so it becomes a lagged averaged of the state of the ICM cells. An explicit lifetime has little effect since the activity of the FGF faded over time since the cells descended down the wells representing the PrE and Epi states, and consistent with the phenomena of commitment, the FGF level was never so high to destabilize these states in the late blastocyst. Thus the topography effectively imposes a limited time window on FGF activity. See Fig S5A. The external FGF is still assumed to act instantaneously, we could model a signal transduction time, but there is no data to constrain it.

4. I hope that the authors can resolve these issues or make it crystal clear within their modelling framework.

We added a new discussion paragraph to pose and resolve the question raised in items 2-3.

Reviewer 2

To set the scale for the data under discussion from Ref 11, we show our own images of an embryo at two time points, the first comparable to Ref 11. The ICM is a very confined space, so a small amount of jiggling will randomize the FGF as will a bit of diffusion.

© 2024. Published by The Company of Biologists under the terms of the Creative Commons Attribution License (https://creativecommons.org/licenses/by/4.0/).

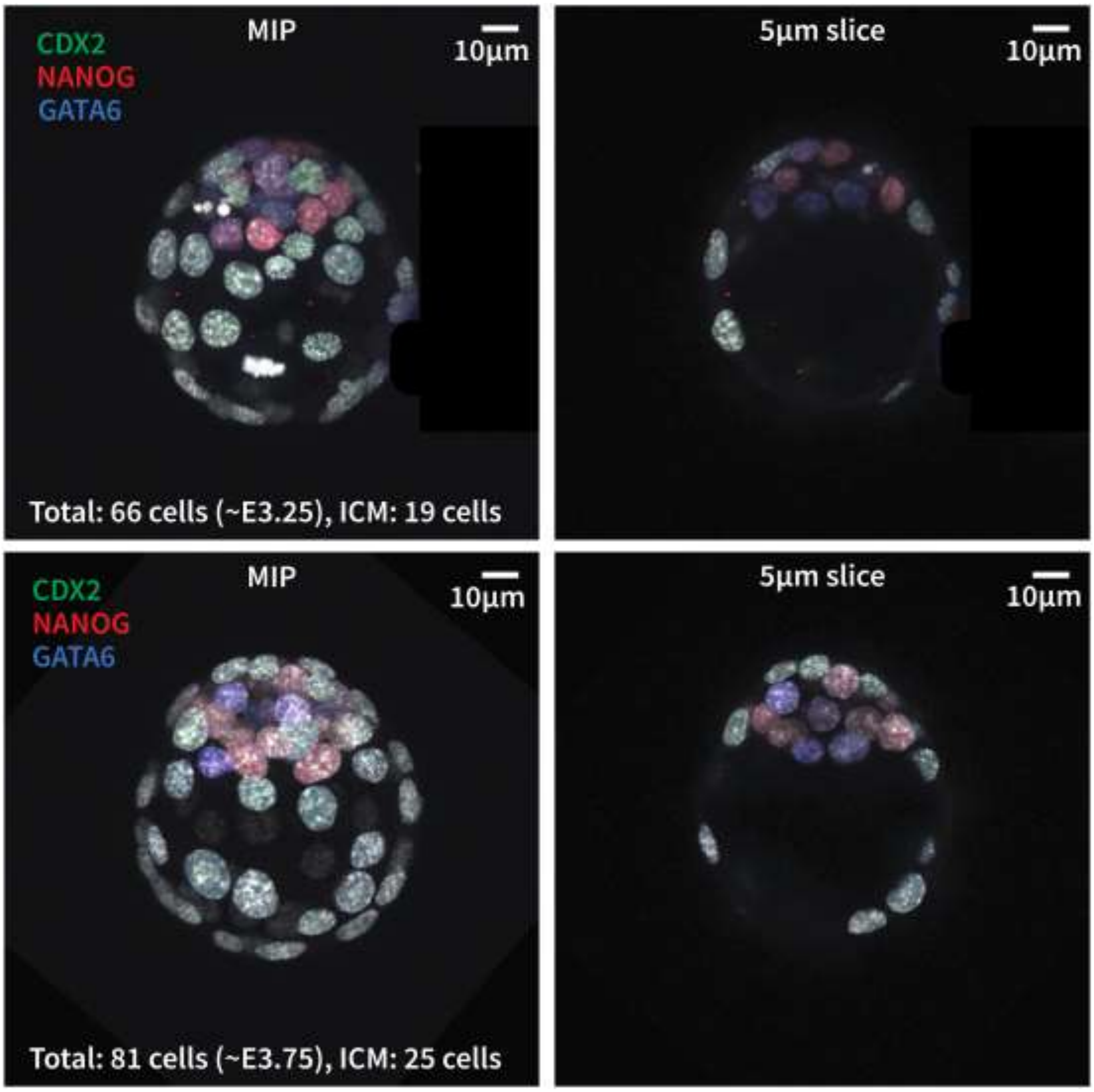

1. *Is the 25 minute estimate the change in vornoi neighbors of every cell? Or is there on average one change in the adjacency matrix every 25 minutes across the whole embryo? Can the data for neighbor exchanges be presented? I believe this is crucial.*

We are now explicit about how the change in Voronoi contacts is done in the Materials and Methods. The 25 min was the average for one contact to change for a typical cell. In the SI, we show several examples of cell movement and report how many Voronoi neighbours change per cell, by several measures. In clarifying our procedure for this estimation, we have also changed it slightly and now report 30 minutes as the change in Voronoi neighbours. The data is all taken from Ref. 11.

2. *From fig1D is certainly seems like cell motion is correlated amongst cells that are closer to each other. Red and green trajectories look highly correlated. Same with cyan and magenta. This would suggest that neighbors dont change as if the system was really “well mixed”.*

We thank the referee for pointing this out. The correlations were because of global movements in the embryo. We now keep the center fixed in time and plot the cell positions for all cells. The 2d projection makes things difficult to see so we now have a plot of the pair distances after 2 hours in Fig S1 and several metrics derived from changes in the adjacency matrix Fig S2 for nine embryos which is another way to see the cell movement.

3. *A more careful time series analysis would be useful. Within the 25 minute window, are the erk fluctuations between cells that are in contact (in the vornoi sense) correlated? Meaning, what is the correlation coeffieicnet between neighboring cells as calculated through a time series analysis within*

© 2024. Published by The Company of Biologists under the terms of the Creative Commons Attribution License (https://creativecommons.org/licenses/by/4.0/).

a 25 minute window? This would shed light on whether space matters.

We show an example of the ERK fluctuations in the SI, Fig S3 and further calculate the correlation coefficient of the time series as suggested in the SI. Note, apart from the cell movement, FGF diffusion and noisy response of ERK contribute to the lack of correlations.

4. Can the Moran statistic please be defined in the manuscript itself? Surely inline equations should be permitted, and used, in such quantitative studies.

We have referenced Materials and Methods in the text and rewritten the paragraph on the Moran statistic. We fear putting in the equation and details will distract rather than elucidate the point.

5. In figure 1b the y axis is the mean of neighbors. This might obscure spatial correlations that might exist between certain pairs. I realize that this is larger probability distribution, but what does the scatter of c:n look like of a cell and its neighbors? Is it bimodal? Some, or few, neighbors show significant correlation and most dont? That would suggest that the mean isn't the right statistics to use.

To avoid confusion, we now say "Nearby cells" rather than Neighbors in Figure 1b as these are not Voronoi neighbors but cells within some fixed distance. To the referee's point, we didn't find any such pattern in the neighbors. We now explicitly do the correlation of the time series for each neighbor in the SI Figure 2 rather than averaging over neighbors.

6. With regards to the lineage analysis. Could the authors show us an example of what the time series looks like right after a division in sister cells? Could the individual trajectories also be shown instead of the 1sd cloud around the mean? Could a correlation between sister times series be performed after the hangover from the mitosis is over — which seems to last on average around 20-30 minutes? It just struck me as to whether the "mean erk" in the lineage analysis is of the c:n ratio itself? Can the authors clarify whether for this lineage analysis they are summing averaging c and n, or is the mean referring to the ratio?

The lineage correlations are not central to this paper and we have moved them to the SI Fig S4, so not emphasize them. We now show an individual trajectory as an example. Sisters are correlated as one would expect, yet we observe a small negative spatial correlation in the population as a whole (Fig1). We have also clarified that we average the ERK C:N in the Figure S4 but have not tried to examine the lineage data further as it is spatial correlations we are concerned with.

7. As a whole, I would like to say that the overall philosophy of making the ansatz that for some reason thinking of the fgf as not spatially resolved, but time-dependent, is valuable. Its accuracy can be judged based on the accuracy of the ensuing geometric models ability to fit the data and predict novel aspects. That all being said, I think the time series analysis perform in figure 1 falls short. More can be done, even if it only furthers the deductions made about the irrelevance of space in the dynamics of differentiation. One important timescale that I believe the authors know, and should either report or estimate, is some kinetic timescale of signaling itself. The 25 minute number must be compared to something else. The something else ought to naively be some timescale associated with signaling itself, which surely is known from other fgf signaling studies. If 25 minutes is slow/large compared to this intrinsic timescale then space matters, potentially. If 25 minutes of the neighbor exchange timescale is fast/small compared to the intrinsic signaling timescale then one can neglect space since the cell so to speak samples multiple signaling sources within one signaling half life.

We now have an SI where we do further analysis of the data as the referee suggests. The relevant time-scale here is the one at which the decisions are taken which is of order a day rather than minutes, hence the 30 minute time is considerably faster than other relevant time scales in the system. We now say this in the discussion. We show time traces for the ERK signal in Fig S3, and observe it's quite noisy so an autocorrelation time to measure the signal transduction is probably not meaningful

8. The beginning of 2.2 can have a little bit more background. Some words associated with saying that there is a categorization of 3 attractors in 2 dimensions and that this has been leveraged by the authors to model differentiation. Just even a little context brings the readers into the world view

© 2024. Published by The Company of Biologists under the terms of the Creative Commons Attribution License (https://creativecommons.org/licenses/by/4.0/).

that this current paper belongs to. In addition, I would ask the authors to explain the reasons that thinking about locality, or lack therefore, and the dimensionality of decision, is valuable as far as the biology goes. To those of us who know local and global, the circle encompassing the fixed points means something. I can imagine to the less mathematically informed reader the word local/global, or the circle construction, doesn't help. Same goes for one vs two dimensional. Can these concepts, which are so immensely powerful/universal/generic be interpreted biologically for that kind of audience? If these ideas are to permeate then the leaders in the field, I believe, must provide interpretation and context!

We have added to the discussion of this section. We feel that more thorough discussion of bifurcation theory belongs elsewhere as it will run the risk of being too pedagogical. Nevertheless, we believe the essential points become clear to biological readers as the paper progresses.

9. For section 2.3: I really wish the authors would pose the incredible value of this section in a manner that could be read and understood by a biologist. The real key fact, in my limited view, is that it is generically observed that the responses of marker genes to signaling perturbations can look entirely non trivial. In particular, the response of the system can be non-monotonic in time. This is a very counter intuitive observation since the loose intuition of your average dev bio person is that commitment increases in time and thus response to signals should always be reducing as the ball falls into a well. This very nice example that the authors show demonstrate that for very good, in fact even more generic reasons, you should expect a non monotonic response functions. In this sense, of course, the words the authors use are entirely spot on. But I would be so much happier if they spend a little real estate making all these connections and analogies to help it and for the biologically oriented reader. Otherwise, sadly, the work of this qualitatively minded community will only be cited by others within, and not without.

As the referee suggests, we have added a paragraph showing how this observation runs counter to ordinary intuition.

10. This sentence in the discussion ".The flip, being a global bifurcation, is ideally suited to transitions where the embryo needs to control the population ratio via morphogen feedbacks " Is deep. More should be said. It is deeply related to the circle construction at the start.

The augmented discussion we did in response to point 7, hopefully elaborates how the flip gives the morphogens time to act and decouples the instability of the progenitor from the choice between the two terminal states. This is the concluding paragraph of the Discussion, so hopefully the logic is clear by this time. Ultimately, we want to encourage well quantified experiments before attempting further elaboration of the model.

Second decision letter

MS ID#: DEVELOP/2023/202467

MS TITLE: A geometrical model of cell fate specification in the mouse blastocyst

AUTHORS: Archishman Raju and Eric Siggia

I am happy to tell you that your manuscript has been accepted for publication in Development, pending our standard ethics checks.

Reviewer 1

Advance summary and potential significance to field

© 2024. Published by The Company of Biologists under the terms of the Creative Commons Attribution License (https://creativecommons.org/licenses/by/4.0/).

The authors have answered the questions I raised in a satisfactory manner. In my opinion the paper makes advances to cell fate specification in mouse.

Comments for the author

The paper is well written and well presented. I believe the paper can be published.

© 2024. Published by The Company of Biologists under the terms of the Creative Commons Attribution License (https://creativecommons.org/licenses/by/4.0/).
